# Cloning and analysis of expression patterns and transcriptional regulation of *RghBNG* in response to plant growth regulators and abiotic stresses in *Rehmannia glutinosa*

**DOI:** 10.1186/s40064-015-0830-0

**Published:** 2015-02-04

**Authors:** Yanqing Zhou, Yonghua Zhang, Jun Wei, Yu Zhang, Jingyun Li, Wanshen Wang, Hongying Duan, Juanjuan Chen

**Affiliations:** College of Life Sciences, Henan Normal University, No.46 Jianshe Road, Xinxiang, 453007, Henan China; Key Laboratory for Microorganisms and Functional Molecules, University of Henan Province, No.46 Jianshe Road, Xinxiang, 453007, Henan China

**Keywords:** *Rehmannia glutinosa*, *RghBNG* gene, Cloning and expression; qRT-PCR; Abiotic stress; Plant growth regulator

## Abstract

*RghBNG*, a gene of unknown function, was cloned from *Rehmannia glutinosa* by reverse transcription PCR and rapid amplification of cDNA ends. The full-length cDNA of *RghBNG* was 548 bp with a282-bp open reading frame. It encoded a polypeptide of 93 amino acids with a predicted molecular weight of 10.5 kDa and a theoretical isoelectric point of 9.25. Bioinformatics analysis indicated that *RghBNG* had no homology to any known plant genes, whereas the RghBNG polypeptide was highly similar to other plant proteins and possessed one conserved B12D protein family functional domain. Phylogenetic analysis revealed that *RghBNG* encoded for a dicot protein. *RghBNG* spatial and temporal expression patterns and responses to abiotic stresses and plant growth regulators were investigated by qRT-PCR. RghBNG transcripts were detected in roots, stems, leaves, petals, receptacles, stamens and pistils with the highest and lowest levels respectively observed in petals and leaves of mature plants. Additionally, RghBNG transcripts were detected at three developmental stages of roots, stems and leaves; the highest levels were observed in roots at seedling stage; Transcript levels changed to varying degrees in different tissues and stages; We also studied the effects of abiotic stress and plant growth regulators in roots and leaves. *RghBNG* expression was significantly increased (*p* < 0.01) by chromium, gibberellic acid and NaCl, with the highest levels induced by chromium stress; In contrast, 6-benzyladenine reduced expression. These results strongly suggest that *RghBNG* is involved in *R. glutinosa* growth, development and response to plant growth regulators and abiotic stresses.

## Introduction

Abiotic stresses reduce average yields of most crops by more than 50% (Wang et al. 2003; Bhatnagar-Mathur et al. 2008). Drought, salinity and extreme temperature are among the major abiotic stresses, hampering plant growth and productivity and frequently cause a series of morphological, physiological and biochemical changes (Mehrotra et al. 2014). In addition, heavy metal contamination of soil and water is a global problem giving rise to crop yield losses and having hazardous effects on human health when these metals enter the food chain (Vernay et al. 2007). Because plant vulnerability to such abiotic stresses is a serious threat to the entire plant ecosystem, molecular and genetic mechanisms of abiotic stress tolerance in plants are of great scientific interest (Alexandra et al. 2013). Plants respond and adapt to stress conditions with an array of biochemical and physiological changes, many of which are regulated by stress-responsive gene expression. The regulation of these abiotic stress responses to generate stress resistant plants is of great importance to plant growers (Sarika and Aaron 2014). One important strategy related to this goal is the development of stress-tolerant transgenic plants (Tuli et al. 2010). To develop such plants, stress-related plant genes must be identified, characterized and tested in plant systems for their potential use in commercially important crops (Kumar et al. 2013; Quaggiotti et al. 2007). *Rehmannia glutinosa* (*Scrophulariaceae*) is a well-known medicinal plant widely cultivated in Asian countries such as China, Korea, Japan, Vietnam and others. The tuberous roots are used to treat fever, nervous conditions, diabetes and hypertension, to strengthen liver function, and for hematopoietic, immuno-enhancing, and tonic purposes (Kim et al. 2012; Sun et al. 2010). Because root yield and quality are limited by various stresses. the incorporation of some stress-tolerant genes is needed to improve *R. glutinosa* resistance. The effects of several abiotic stresses, such as temperature, water, NaCl, Paraquat and choline chloride have been previously reported for *R. glutinosa* (Chung et al. 2006; Jia et al. 2009; Zhao et al. 2007; Piao et al. 2008). The NaCl- and waterlogging stress-intolerant *Aux/IAA* gene from *R. glutinosa* has also been recently described (Wang et al. 2013). Nevertheless, the effects of many other abiotic stresses and the functions of other stress-related genes remain unknown in *R. glutinosa*. In the present study, we therefore isolated the *RghBGN* gene from *R. glutinosa*. To elucidate the role of *RghBGN* in *R. glutinosa* growth, development, and stress responses, we investigated its expression patterns in the presence and absence of abiotic stresses and plant growth regulators.

## Results

### Cloning and sequencing of *RghBNG*

**Figure 1:**
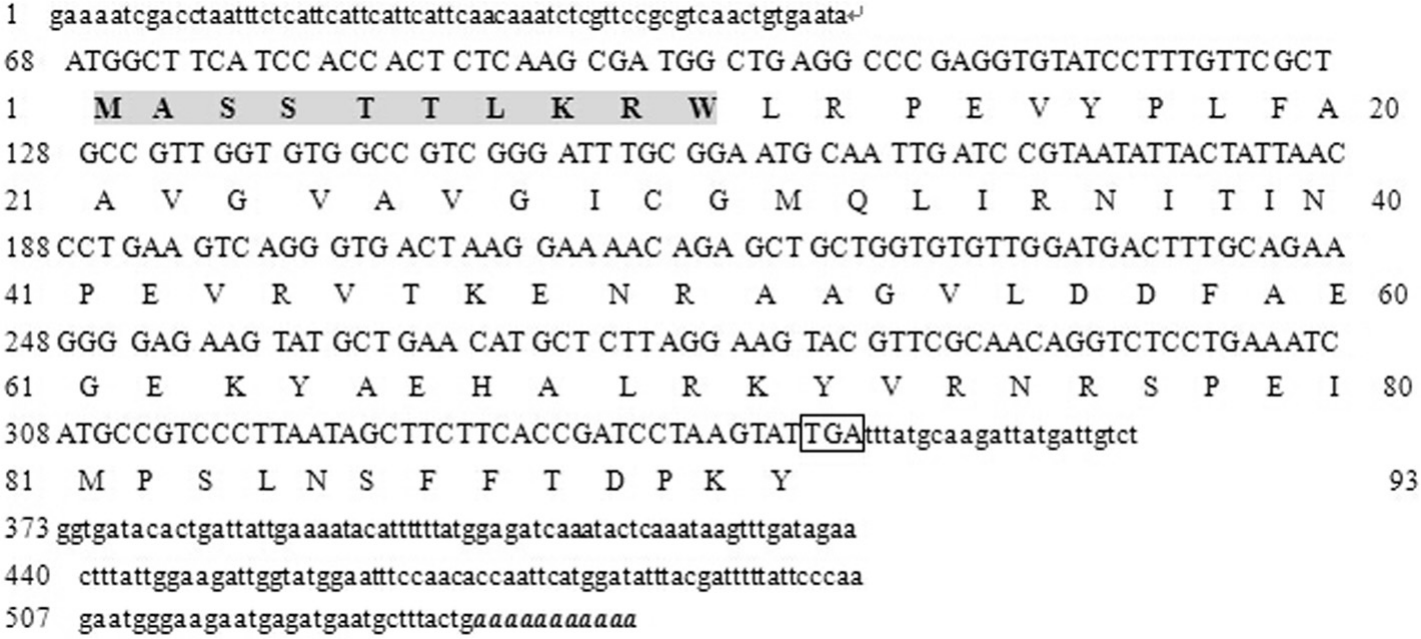
**Nucleotide sequence of the full-length cDNA and deduced amino acid sequence of** ***RghBNG*** **.** Numbers on the left and right correspond to nucleotide and amino acid sequences, respectively. In the nucleotide sequence, the stop codon is boxed and the polyadenylated tail is indicated in bold italic letters. In the amino acid sequence, the B12D superfamily motif is shaded and in bold.

One 221 bp cDNA fragment (middle fragment) was amplified by RT-PCR with the primer pair P1, cloned and sequenced. Its 3′-end product and 5′-end product were generated by 3′-RACE and 5′-RACE, respectively. After both were sequenced, three fragments were aligned linked together to give a 548 bp full-length cDNA known as *RghBNG* (GenBank Accession No.JX290370) including a 5′-untranslated region of 68 bp and 3′- untranslated region of 199 bp. Its 282-bp open reading frame (ORF) was searched by ORF Finder (Figure 1). *RghBNG* gene was amplified by RT-PCR with primer pair P4.

### Bioinfomatics analyses of *RghBNG* gene

**Figure 2:**
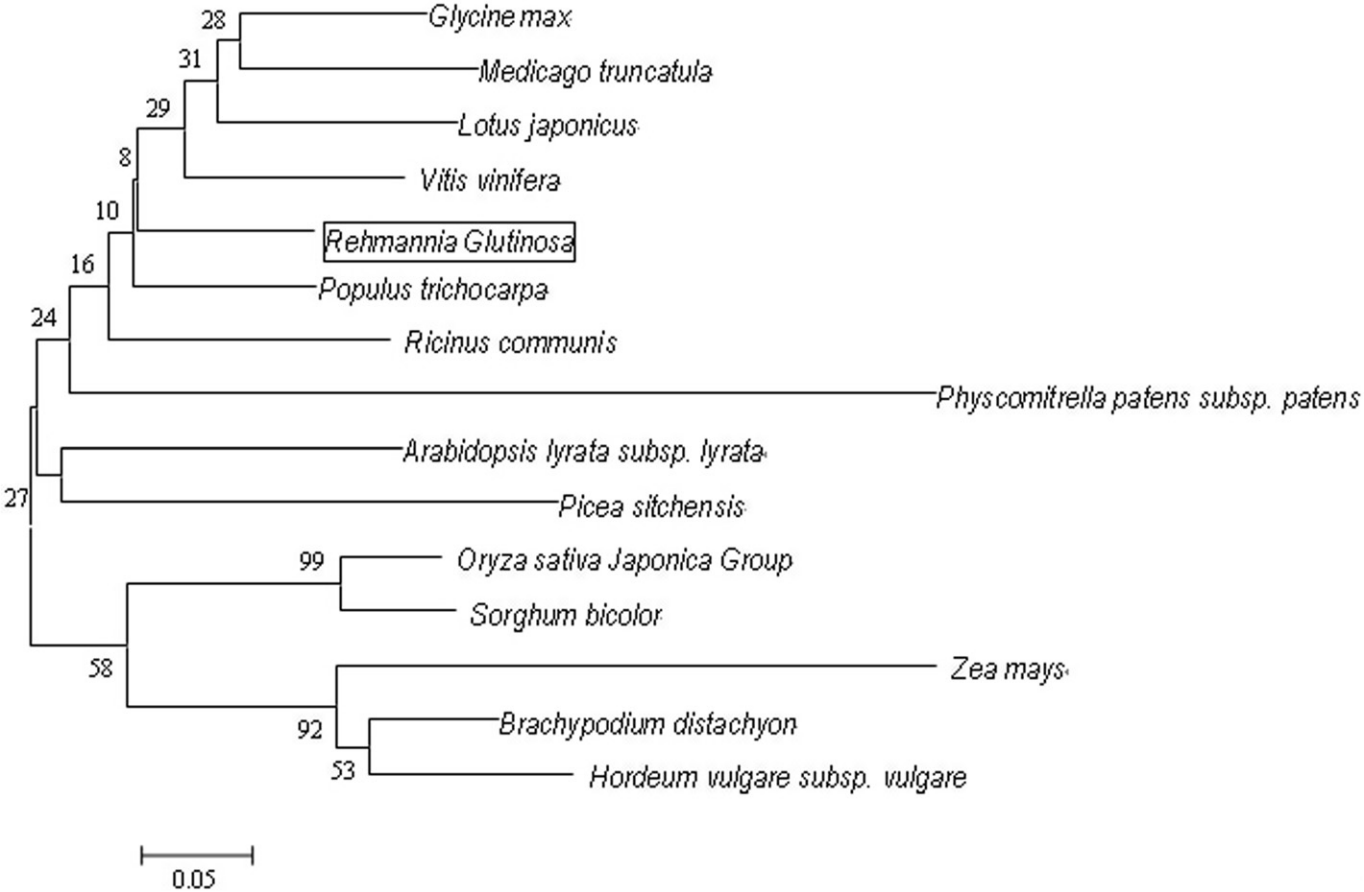
**A molecular phylogenetic tree of RghBNG and related proteins generated by neighbor-joining method using MEGA 4.0.**The unrooted phylogenetic tree was generated based on the alignment of amino acid sequences from 14 plant species: *Populus trichocarpa* (XP_002318014.1; 86% similarity to the RghBNG ), *Glycine max* (XP_003527039.1; 84%), *Ricinus communis* (XP_002515002.1; 82%), *Lotus japonicus* (AFK42697.1; 81%), *Medicago truncatula* (ACJ84101.1; 79%), *Arabidopsis lyrata subsp* (XP_002875880.1; 79%), *Vitis vinifera* (XP_002283743.1; 78%), *Brachypodium distachyon* (XP_003557311.1; 72%), *Picea sitchensis* (ABK24106.1; 70%), *Oryza sativa Japonica Group* (NP_001057280.1; 70%), *Sorghum bicolor* (XP_002438146.1; 70%), *Hordeum vulgare subsp. Vulgare* (BAK01669.1; 70%), *Zea mays* (ACN31786.1; 59%) and *Physcomitrella patens subsp. Patens* (XP_001779363.1; 58%). Numbers above nodes are bootstrap support percentages based on 1,000 replicates. The scale bar indicates evolutionary distance of amino acid substitutions per position. RghBNG from *Rehmannia glutinosa* is boxed.

Bioinfomatics analyses indicated that *RghBNG* has no homology to any known gene in NCBI at the cDNA level. However, its open reading frame may encode a protein of 93 amino acid residues in length (Figure 1).The protein is composed of 20 amino acids, of which alanine accounts for 9.7%, both arginine and valine for 8.6%, each of the rest for 1.1%-7.5%, alkaline amino acids for14.0% and acidic amino acids for10.8%, in which hydrophilic amino acids and hydrophobic amino acids are evenly distributed, and there are no signal peptide but four phosphorylation sites including 1 Ser site, 1 Thr site and 2 Tyr sites. Its secondary structure is composed of four parts such as α-helixes (56.99%), random coils (27.96%), β-sheets (9.68%) and β-turns (5.38%). Its theoretical isoelectric point (pI), molecular weight and molar extinction coefficient are, 9.25, 10.52 and 56.74, respectively. Furthermore, it has one conserved functional domain of B12D superfamily (Figure 1). Moreover, similarity analyses by the NCBI-blastp showed that the RghBNG protein had a high degree of similarity (58%--86%) to some unknown functional proteins from 14 known species (Figure 2). Meanwhile, it also has another high degree of similarity (>72%) to B12D proteins such as 80% to *Beta vulgaris* (CAK22419.1), 79% to *Camellia sinensis var. assamica* (Mast.) Kitam (AEC10990.1), 79% to *Arabidopsis thaliala* (NP_190397.1), 74% to *Ipomoea batatas* (AAD22104.1), 73% to *Castanea sativa* (AAL17696.1) and 72% to *Wolffia arrhiza* (ADB08699.1).

To investigate the phylogenetic relationship of RghBNG with other known homologous proteins, the amino acid sequences of available unknown functional proteins from 14 known species were used to construct the phylogenetic tree (Figure 2). This analysis showed that RghBNG clusters with *Glycine max*, *Medicago truncatula*, *Lotus japonicus* and *Vitis vinifera* with a support value of 29, and that RghBNG protein is attributed to dicots (Figure 2), which is in accordance with APG classification system.

### Expression pattern of *RghBNG* in different *Rehmannia glutinosa* tissues and stages

**Figure 3:**
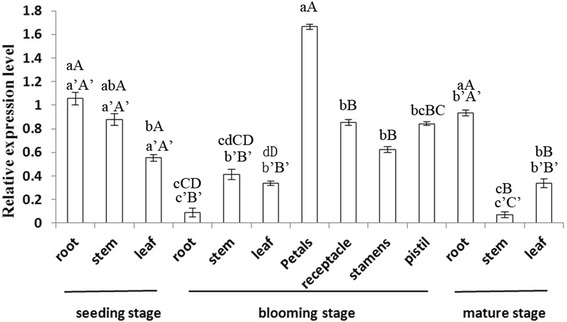
**Expression pattern of the** ***Rehmannia glutinosa RghBNG*** **gene in different tissues at three different developmental stages as determined by quantitative real-time PCR.** Expression levels are normalized relative to those of *TIP41*, which was used as an internal control. Data are means ± SE of three replicates. a-d and A-D in relative (spatial) expression level indicate the significance at 0.05 and 0.01, respectively; a’-c’and A’-C’ in relative (temporal) expression level indicate the significance at 0.05 and 0.01, respectively.

To determine the spatial expression pattern, cDNA was synthesized from three tissues of the plants at seedling stage, seven ones at full blooming stage and three ones at mature stage of *Rehmannia glutinosa*. It was found that RghBNG mRNA was expressed in all the tissues tested, with the strongest signal in root and the significant difference between root and leaf at seedling stage (α = 0.05); with the strongest signal in petal and the extremely significant one between petal and each of others, the one between receptacle or stamen and root or stem or leaf, the one between leaf and pistil at full blooming stage (α = 0.01); with the strongest one in root, and the extremely significant one between root and stem or leaf (α = 0.01), and the significant one between stem and leaf (α = 0.05) at mature stage (Figure 3). All qRT-PCR products of RghBNG gene was confirmed by sequencing.

The temporal expression profile of RghBNG RNAs during root, stem and leaf developments was determined by qRT-PCR. Their expression levels at three stages were summarized in Figure 3. It was found that RghBNG mRNA was expressed at all the stages tested, with the strongest signal at seedling stage, the extremely significant difference between blooming stage and seedling stage or mature stage (α = 0.01) and the significant one between seedling stage and mature stage (α = 0.05) for root; with the strongest signal at seedling stage and the extremely significant one between one and each of others among three ones (α = 0.01) for stem; with the strongest one at seedling stage, and the extremely significant one between seedling stage and blooming one or mature one (α = 0.01) for leaf. All qRT-PCR products of RghBNG gene was confirmed by sequencing.

**Figure 4:**
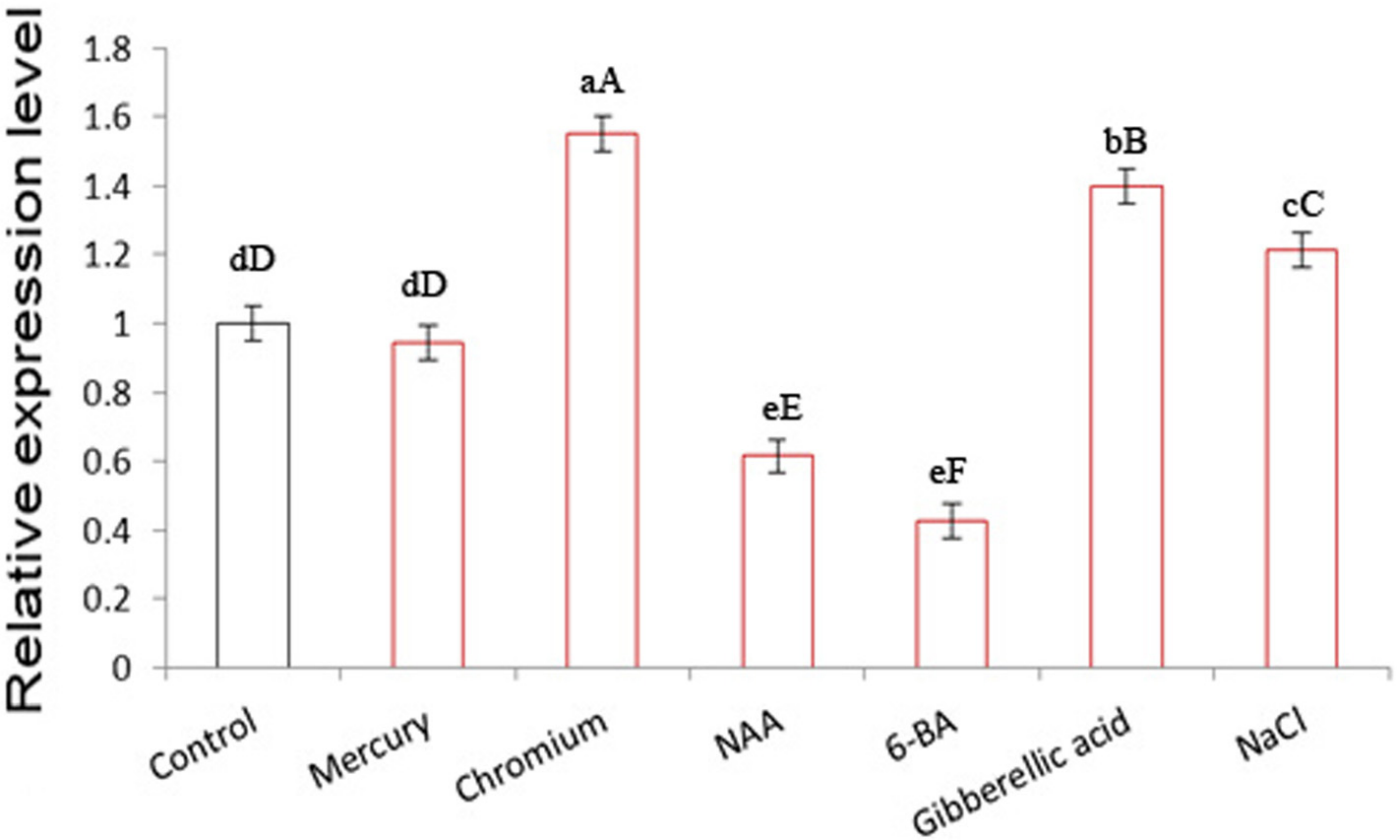
**Expression of the** ***Rehmannia glutinosa RghBNG*** **gene in roots of plants exposed to chromium, mercury, NaCl, gibberellic acid, 1-napthalene acetic acid (NAA) or 6-benzyladenine (6-BA).** Following treatments, total RNAs were isolated, and RghBNG expression levels were monitored by quantitative real-time PCR. Error bars indicate standard deviation values of three independent analyses. a-c and A-C in relative expression level indicate the significance at 0.05 and 0.01, respectively.

### Gene transcription regulation of the *RghBNG* gene

**Figure 5:**
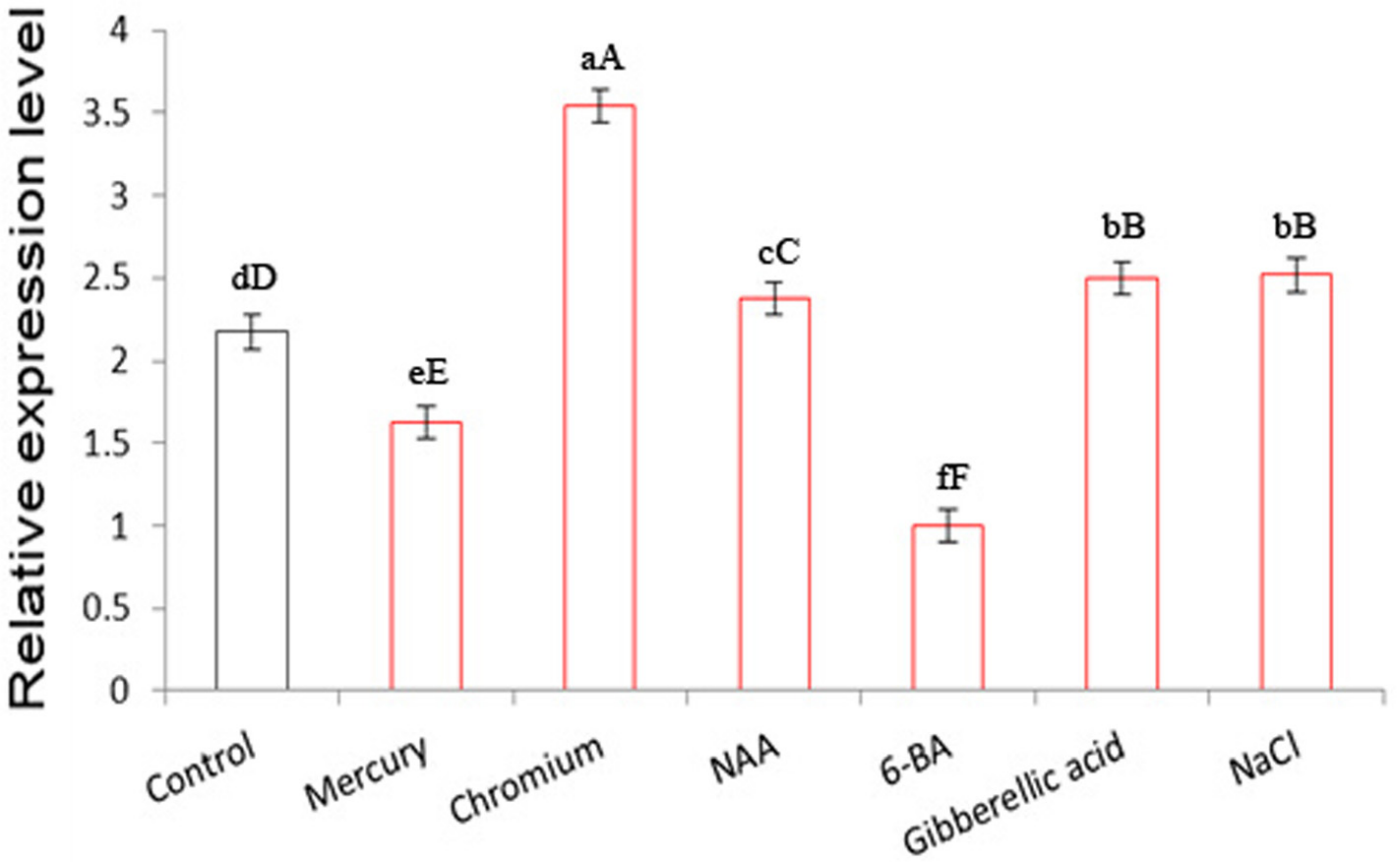
**Expression of the** ***Rehmannia glutinosa RghBNG*** **gene in leaves of plants exposed to chromium, mercury, NaCl, gibberellic acid, 1-naphthalene acetic acid (NAA) or 6-benzyladenine (6-BA).** Following treatments, total RNAs were isolated, and RghBNG expression levels were monitored by quantitative real-time PCR. Error bars indicate standard deviation values of three independent analyses. a-c and A-C in relative expression level indicate the significance at 0.05 and 0.01, respectively.

In order to investigate if the expression of the *RghBNG* gene is associated with the abiotic stress and plant growth regulators-induced defense mechanism in *Rehmannia glutinosa*, the expression pattern of the *RghBNG* was examined in young *Rehmannia glutinosa* plants subjected to six factors such as chromium, mercury, NaCl, Gibberellin, NAA and 6-BA treatments by qRT-PCR (Figures 4 and 5). For root as shown in Figure 4, compared to control, Chromium, exogenous Gibberellin and NaCl resulted in different increases in *RghBNG* transcript levels with the highest one induced by chromium, whose ones were extremely significant (α = 0.01), and that NAA and 6-BA down-regulated *RghBNG* transcript levels, whose ones were extremely significant (α = 0.01). However, mercury did not significantly decrease the expression level (α = 0.05). For leaf as shown in Figure 5, Chromium, NAA, Gibberellin and NaCl also resulted in different increases in *RghBNG* transcript levels with the highest one under chromium stress, whose ones were extremely significant (α = 0.01), while mercury and 6-BA decreased *RghBNG* transcript levels, whose ones was also extremely significant (α = 0.01) compared to control.

## Discussion

### Cloning, sequencing, and structural analysis of *RghBNG*

In this study, we cloned the full-length sequence of the *RghBNG* gene, deduced its amino acid sequence, and analyzed the structure of the encoded protein. The *RghBNG* gene was obtained from *R. glutinosa* using RT-PCR and RACE technology. No homology was found between the *RghBNG* cDNA sequence and that of any other plant species gene, but considerable similarity was uncovered between the amino acid sequence of the deduced RghBNG protein and proteins from other species, which could be divided into two groups: B12D proteins, and proteins of unknown function. In addition, RghBNG protein possesses the conserved structure domain of B12D protein family, B12D superfamily,(Walker et al. 1992, Figure 1), belonging to one kind of subunits of the NADH: ubiquinone oxidoreductase (Complex I) in mitochondrial membrane (Gabaldón et al. 2005; Zhang et al. 2012).

### The roles of other known B12D protein genes

The B12D gene was first isolated from barley (Aalen et al. 1994) and was initially characterized as a transcript related to barley aleurone and embryo development (Steinum et al. 1998). Several B12D protein-like protein genes have been subsequently cloned and their roles have been studied. For example, B12D protein has been shown to be localized in peroxisomes of Arabidopsis and rice (Kaur and Hu 2011), and GA3 up-regulates B12D protein gene expression in imbibed barley embryoless grains (Aalen et al. 1994). However, abscisic acid (ABA) suppresses B12D expression in imbibed barley embryoless grains (Steinum et al. 1998), while ABA and 20% mannitol inhibit its expression in barley embryos (Aalen et al. 1994). In another study, B12D protein was specifically expressed by the induction of ABA (Aalen and Steinum 2001). A B12D-like protein of unknown function was found to be constitutively expressed in Arabidopsis seedlings, roots, leaves, inflorescences, flowers and siliques at different developmental stages (Zhu et al. 2001). A soybean B12D protein gene (Glyma11g37370.1) has been observed to increase the female index of soybean cyst nematode (Matthews et al. 2013). The C832 (DT045059) gene, which has been found to be differentially expressed between wheat near-isogenic lines Chisholm-T and Chisholm-S under Al stress, shows similarity with known putative B12D protein genes in GenBank and TIGR databases (Guo et al. 2007). Plant-specific B12D proteins of unknown function are strongly down-regulated in root tissues of wheat genotypes with distinct responses to water deficit (Ergen et al. 2009). A B12D protein encoded by TDF41 (NP_001057280) is induced by sorbitol, and indirectly promotes plantlet regeneration frequency of rice calli (Feng et al. 2011). An abiotic stress-regulated cDNA clone (CD725202) from *Pennisetum* encodes proteins involved in cellular homeostasis, including a B12D-like protein that protects plants from stress-induced damages and facilitates the establishment of cellular homeostasis (Mishra et al. 2007). A cDNA sequence (CdR311) with identity to the mRNA sequence of a *Zea mays* B12D-like protein (AY104072) is up-regulated in roots of Bermuda grass growing under petroleum stress, but its biological activity remains unknown (Peña-Castro et al. 2006), B12D proteins may be involved in general functions such as signal transduction, disease resistance, cell growth, and differentiation (Vinod et al. 2010). A B12D protein cDNA (BP947551) showed 100% transcript variation in NaCl-treated leaves and roots of *Burma mangrove*, making it a perfect candidate as a stress-responsive protein (Miyama et al. 2006). Another B12D-like protein gene (BP945437) has been discovered to be highly repressed in salt-treated *Burma mangrove* roots (Miyama and Hanagata 2007). *Castanea sativa* B12D-like protein mRNA is differentially expressed upon wounding and infection of chestnut with chestnut blight fungus (Schafleitner and Wilhelm 2002). B12D proteins also have been inferred to participate in ATP synthesis and energy metabolism (Gabaldón et al. 2005; Zhang et al. 2012).

### Spatial and temporal expression of *RghBNG*

The roles of *RghBNG* in plant development were examined by characterizing its spatial and temporal expression patterns during *R. glutinosa* growth and development by qRT-PCR. RghBNG mRNA transcripts were detected in all tested tissues and organs, but the transcript levels varied. The highest levels were clearly detected in petals at the full-blooming stage and roots at both seedling and mature stages (Figure 3), demonstrating that *RghBNG* may play an important role in *R. glutinosa* growth and development.

### Transcriptional expression of *RghBNG* in response to abiotic stresses and plant growth regulators

Plants are frequently exposed to many forms of stress. Plant adaptation to stress has been suggested to be mediated by both preexisting and induced defense mechanisms. Plant signaling pathways are driven by plant growth regulators and reactive oxygen species. Phytohormones generate a signal transduction network that leads to a cascade of events responsible for plant adaptation to external conditions (Al-Momany and Abu-Romman 2014). To assign possible gene physiological functions, detailed expression analysis of the target gene is an important and necessary step (Pérez-Torres et al. 2009). In our study, *RghBNG* responses to abiotic stresses and plant growth regulators were investigated by qRT-PCR. The *RghBNG* gene was induced by GA3 and NaCl, in agreement with previous studies of B12D proteins (Steinum et al. 1998; Aalen et al. 1994; Aalen and Steinum 2001; Miyama et al. 2006; Zhang et al. 2012). Furthermore, our results revealed that the *RghBNG* gene significantly responded to abiotic stress induced by chromium and plant growth regulators such as NAA and 6-BA (Figures 4 and 5). These findings reveal that RghBNG proteins possessed some known roles for B12D proteins such as GA3 and NaCl induction and novel roles for B12D proteins (one group) or the proteins of unknown function (the other group) mentioned above, for example, responses to Cr, Hg, NAA and 6-BA.

## Conclusions

A full-length cDNA of the *RghBNG* gene from *R. glutinosa* was cloned and characterized, and its spatial and temporal expression profiles and abiotic stress and plant growth regulator-responsive patterns were determined. This study has provided essential molecular data that can be used in follow-up studies to elucidate the biological roles of *RghBNG* in growth, development, and stress and plant growth regulator-responses of *R. glutinosa*.

## Materials and methods

### Plant materials and treatment conditions

Sterile 25-day-old plants of *R. glutinosa* f. *hueichingensis* ‘Wen85-5′ were grown in hormone-free Murashige-Skoog (MS) media or transplanted into flower pots in a greenhouse to obtain full-blooming plants and mature plants (Zhou et al. 2009). Leaf sample were collected for gene cloning. For expression pattern analysis, samples of fresh leaves, stems, roots, petals, receptacles, stamens and pistils were collected from *R. glutinosa* plants during corresponding vegetative growth, full-blooming and early senescence phases. To investigate gene expression regulation under abiotic and hormonal stresses, 1.5-cm-long shoots with two leaves from 25-day-old plants, five shoots per treatment, were inserted and maintained for 20 days in a 450-mL glass bottle containing MS medium either lacking (control) or supplemented with one of the following impact factors: 200 μM NAA, 500 μM 6-BA, 150 μM K_2_Cr_2_O_7_ or 200 μM HgCl_2_. At the end of the treatment period, five control plants each were sprayed every other hour for 48 h either with a solution of 100 μM GA3 or 250 mM NaCl, and leaf and root samples were then collected. The experiment was repeated three times in a greenhouse. All samples were immediately frozen in liquid nitrogen after collection and stored at −80°C until further analysis.

### Total RNA extraction and first-strand cDNA preparation

Total RNA was extracted from leaf samples using an RNA kit (Lifefeng Biotechnology Co., Shanghai, China). RNA concentration and purity were estimated spectrophotometrically based on absorbance at 260 and 280 nm. First-strand cDNA was synthesized from 1 μg total RNA, which was reversely transcribed using M-MLV reverse transcriptase with oligo (dT)_15_ as a primer in a 10-μL reaction volume (Takara, Japan).

### Cloning of *RghBNG* cDNA

The middle region of the *RghBNG* gene was amplified using first-strand cDNA as a template with primer pair P1 (Table 1). The amplification reaction was performed in a total volume of 25 μL using a CodeDR011 PCR amplification kit (Takara). Amplification conditions were as follows: 94°C for 5 min followed by 30 cycles of 94°C for 30 s, 46°C for 1 min and 72°C for 1 min, with a final extension of 72°C for 10 min. *RghBNG* 5′ and 3′ ends were cloned with primer pairs P2-P2′ and P3′-P3 using 5′-and 3′-Full RACE kits (Takara) according to the manufacturer’s instructions. The full-length cDNA of the *RghBNG* gene was obtained by aligning the three cDNA fragments using DNAMAN4.0 software. The ORF was determined with ORF Finder and cloned by RT-PCR with primer pair P4 (Table 1), which was designed based on the ORF nucleotide sequence. All amplicons were separated, excised, purified, ligated, transformed and sequenced following Al-Momany and Abu-Romman (2014), except that we replaced the T vector and host used in that study with pMD19-T vector and *Escherichia coli* DH5α.

**Table 1:** **Primers used in this study**

**Primer pair**	**Primer sequence (5′ → 3′)**	**Description**
P1	F: GGTCTAACRGCRTCWYTGTT	Gene cloning
	R: AATBGGRACYAAYARCARCATA	
P2	OuterF: GCTGGGCTGACTCCACCTACTCT	Gene cloning
	InnerF: CGCTTGCCCAATTGTTTGTGGCC	
P3	OuterR: GACTTCAGGGTTAATAGTAAT	Gene cloning
	InnerR: CGCAAATCCCGACGGCCACAAAC	
P2′	OuterR:TACCGTCGTTCCACTAGTGATTT	Gene-specific primer for 3′ RACE
	InnerR: CGCGGATCCTCCACTAGTGATTTCACTATAGG	
P3′	OuterF: CATGGCTACATGCTGACAGCCTA	Gene-specific primer for 5′ RACE
	InnerF: CGCGGATCCACAGCCTACTGATGATCAGTCGATG	
P4	F: CGCGGATCCAAAATCGACCTAATTTCTC	Gene-specific primer for ORF
	R: CCGCTCGAGCAGTAAAGCATTCATCTC	
P5	F: GTTGGTGTGGCCGTCGGGATTT	Gene-specific primers for real-time qPCR
	R: AGCATACTTCTCCCCTTCTGCAAAG	
P6	F: TGGCTCAGAGTTGATGGAGTGCT	Gene-specific primers for *TIP41*
	R: CTCTCCAGCAGCTTTCTCGGAGA	

### Bioinformatics analyses

The *RghBNG* nucleotide sequence was aligned using BLAST on the NCBI website. The ExPASy proteomics server (http://www.expasy.ch/tools/protparam.html) was used to determine the amino acid sequence, molecular weight, isoelectric point and other physicochemical properties of the RghBNG protein. The Expert Protein Analysis System proteomics server (http://www.expasy.org/) and the Predict Protein server (http://www.Predictprotein.org/) were used to predict the structure and function of the encoded protein. Phylogenetic trees were constructed by the neighbor-joining method with 1,000 bootstrap replicates using MEGA 4.0 software.

### Analyses of *RghBNG* gene expression and transcriptional regulation

We used qRT-PCR to monitor *RghBNG* transcript levels in tissues and organs of *R. glutinosa* plants at three developmental phases as well as leaves and roots subjected to abiotic and hormonal stress treatments. PCR experiments were carried out using the first-strand cDNAs as templates with primer pair p5 (Table 1) as described above. As a control, *TIP41* was also amplified by RT-PCR using primer pair P6 (Table 1). qRT-PCR was performed on an ABI 7500 Real-Time PCR instrument (Cwbiotech, Beijing, China) in 20-μL reaction volumes comprising 1 μL of cDNA template, 10 μL of 2× UltraSYBR mixture (with ROX), 1 μL of each primer (5 μM), and 8 μL ddH_2_O. PCR conditions were as follows: 95°C for 10 min followed by 40 cycles of 95°C for 15 s, 60°C for 1 min and 72°C for 5 min. The fluorescent product was detected in the last step of each cycle. Amplicons were diluted 16-fold and used to generate a calibration curve for determination of the PCR amplification efficiency of each gene. After acquiring *Ct* values of *RghBNG* and *TIP41*, relative mRNA expression (relative quantity) of target genes was determined with the formula described in Liu et al. (2012). Results were presented as means ± standard error (SE) of data from triplicate replicates. Data were additionally analyzed by one-way analysis of variance and the least significant difference test in SPSS 13.0.

## Abbreviations

*RghBNG*: *Rehmann glutinosa*. f. *hueichingensis* binary novel gene
NAA: 1-Naphthaleneacetic acid
6-BA: 6-Benzylaminopurine
RT-PCR: Reverse transcription PCR
qRT-PCR: Quantitative real time PCR
RACE: Rapid amplification of cDNA ends
GA3: Gibberellic acid
MS: Murashige and Skoog medium
